# Combination of the anti-CD30-auristatin-E antibody-drug conjugate (SGN-35) with chemotherapy improves antitumour activity in Hodgkin lymphoma

**DOI:** 10.1111/j.1365-2141.2008.07146.x

**Published:** 2008-05-08

**Authors:** Ezogelin Oflazoglu, Kim M Kissler, Eric L Sievers, Iqbal S Grewal, Hans-Peter Gerber

**Affiliations:** 1 Departments of Preclinical TherapeuticsBothell, WA, USA; 2 Clinical, Seattle Genetics Inc.Bothell, WA, USA

**Keywords:** SGN-35, antibody-drug conjugate, ABVD, gemcitabine, Hodgkin lymphoma

## Abstract

The antibody-drug conjugate (ADC) cAC10-vcMMAE consists of the tubulin inhibitor monomethyl auristatin E (MMAE) conjugated to the chimeric anti-CD30 monoclonal antibody cAC10. This ADC potently interferes with the growth of CD30-positive haematological tumours, including Hodgkin lymphoma (HL) and anaplastic large-cell lymphoma. This study found improved antitumour activity in a preclinical model of HL when SGN-35 was combined with chemotherapeutic regimens such as ABVD (doxorubicin, bleomycin, vinblastine and dacarbazine) or gemcitabine. Improved efficacy was also observed in high tumour burden models, indicating that combining ADCs with chemotherapeutic agents may be advantageous for the treatment of patients with relapsed or refractory HL.

CD30 is a member of the tumour necrosis factor receptor superfamily and displays limited expression on normal tissues, restricted to activated B and T lymphocytes. CD30 is also expressed in malignancies, including Hodgkin lymphoma (HL), anaplastic large-cell lymphoma (ALCL), immunoblastic lymphoma, multiple myeloma, adult T-cell lymphoma leukaemia, germ-cell malignancies and thyroid carcinoma (reviewed in Younes & Kadin, 2003). As CD30 is undetectable on parenchymal cells in healthy tissues or resting monocytes and lymphocytes, it represents an ideal target for immunotherapy for the treatment of cancer.

We previously reported potent antitumour effects of the cAC10-vcMMAE (SGN-35) drug conjugate in models of HL and ALCL (Francisco *et al*, 2003). The antibody-drug conjugate (ADC) contains a peptide linker (valine–citrulline) that is subject to cleavage by lysosomal enzymes following internalization into target cells (Francisco *et al*, 2003). Release of monomethyl auristatin E (MMAE) into the cytosol induces G2/M-phase growth arrest and cell death through the induction of apoptosis of CD30-positive tumours (Francisco *et al*, 2003). Administration of SGN-35 to mice carrying ALCL and HL tumours induces tumour regressions (Hamblett *et al*, 2004). The unconjugated cAC10 antibody SGN-30 (Wahl *et al*, 2002) and SGN-35 are both under evaluation in clinical trials for the treatment of CD30-positive haematological malignancies, including ALCL and HL (Schnell & Borchmann, 2006).

Recurrent and refractory HL remains an unmet medical need and new strategies to further improve outcome and to reduce therapy-induced complications are needed. The ABVD chemotherapy regimen, consisting of repeated cycles of doxorubicin, bleomycin, vinblastine and dacarbazine (ABVD), is considered a reasonable standard of care in the first line treatment of HL. ABVD, administered either alone or combined with irradiation therapy, results in 2-year survival rates of >90% [reviewed in (Klimm *et al*, 2005)]. Relapse rates after first line treatment range from 5% for early stage disease to 35% for patients with advanced disease. Patients with relapsed and refractory HL typically receive platinum-based combination chemotherapy salvage regimens followed by high dose chemotherapy and autologous stem cell transplantation (Byrne & Gockerman, 2007). Long-term complications vary by treatment regimen and modality. These complications include infertility, cardiopulmonary toxicity and secondary malignancies, including breast cancer and lymphomas. Gemcitabine is an active and reasonably well-tolerated therapy for the salvage treatment of HL. In relapsed or refractory transplant-naïve patients receiving gemcitabine, a 39% response rate with an overall survival time of 10·7 months in the absence of any overt toxicities was reported (Santoro *et al*, 2000). Therefore, novel treatment options with improved therapeutic indices are needed for the treatment of HL, particularly for the treatment of relapsed or refractory patients. This may be achieved by targeting cytotoxic agents more specifically to tumour cells. The present study demonstrated that combining an ADC selectively targeting a tumour antigen on neoplastic cells with conventional chemotherapy can significantly improve antitumour responses in HL without impacting toxicity.

## Materials and methods

Compounds tested include adriamycin (0·75 mg/kg, q4dx3, i.v.; Bedford Labs, Bedford, OH, USA), bleomycin (6 mg/kg, q4dx3, i.p.; Sicor Pharmaceuticals, Irvine, CA, USA), vinblastine (0·01 mg/kg, q4dx3, i.p.; Bedford Labs), dacarbazine (Sicor Pharmaceuticals), gemcitabine (120 mg/kg, q4dx3, i.p.; Eli Lilly and Company, Indianapolis, IN, USA) or vinorelbine (4 mg/kg, q5dx3, i.p.; Pierre Fabre Pharmaceuticals, Parsippany, NJ, USA). The L540cy tumour model and SGN-35 were described previously (Francisco *et al*, 2003). To determine the maximum tolerated dose (MTD) of ABVD and gemcitabine, body weights of severe combined immunodeficient (SCID) mice were assessed daily after treatment with increasing amounts of drugs. The criteria defining the MTD were ≥20% decrease in body weights or other signs of morbidity during the entire treatment followed by a 2-week recovery period. Tumour quadrupling or tripling times were chosen as time-to-endpoint (TTE), which were determined by using the nonlinear regression analysis for exponential growth of each individual tumour growth data set from each experimental animal. The tumour quadrupling time was calculated based on the tumour volume at the beginning of treatment. Animals that did not reach this endpoint were assigned a TTE-value equal to the last day of the study. %TGD (tumour growth delay) reflects the delay in reaching TTE relative to control-treated tumours, which was determined by using the formula: %TGD = [(*T*−*C*)/*C*] × 100, where *T* and *C* are the median times in days for treated and control groups, to reach TTE, using the start of treatment as day 1. Statistical analysis and graphic presentations were conducted using Prism (GraphPad) software for Windows 3.03 software. Tumour growth curves show group mean tumour volumes as a function of time. Data shown are from one representative of two independent experiments. The Logrank test was used to analyse the significance of the differences between TTE of treated and control tumour groups, with differences deemed significant (*) at 0·01 ≤ *P* ≤ 0·05, and highly significant (**) at *P* ≤ 0·01.

## Results and discussion

**Fig 1:**
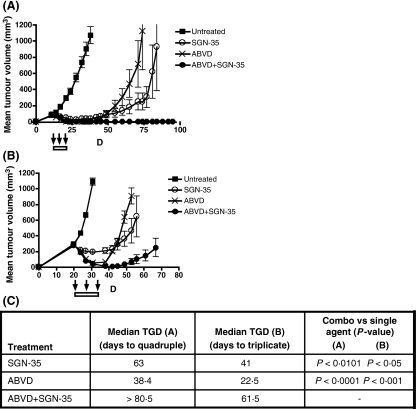
Antitumour activity of SGN-35 in combination with ABVD on subcutaneous L540cy Hodgin-lymphoma (HL) tumours in severe combined immunodeficient (SCID) mice. SCID mice were implanted with L540cy HL cells in the right flank. Groups of mice (9–10/group) were untreated or received SGN-35 (1 mg/kg, q4dx3, i.p.) and/or ABVD [Adriamycin (0·75 mg/kg, q4dx3, i.v.), Bleomycin (6 u/kg, q4dx3, i.p.), Vinblastine (0·01 mg/kg, q4dx3, i.p.) and Dacarbazine (15 mg/kg, q3dx4, i.p.)] when tumour size averaged approximately 100 mm^3^ (A) or 300 mm^3^ (B). The onset and duration of treatment is indicated by the bars within the figures. Bars within the graph represent standard deviation. (C) Median delay to a four- or threefold increase in tumour size (days) relative to untreated tumours are shown for individual treatment groups shown in panel A and B respectively.

The ABVD regimen combines the different mechanisms of action of four anticancer agents. Adriamycin and bleomycin both interfere with DNA synthesis and repair, while vincristine prevents the formation of microtubules and dacarbazine is an alkylating agent that blocks cellular replication by forming cross-linkages between DNA strands. The maximally tolerated dose and schedule for each chemotherapeutic regimen was determined in preceding tolerability studies in tumour-free SCID mice as described in the *Methods*. The 1 mg/kg, q4dx3 treatment schedule for SGN-35 was selected based on previous reports demonstrating maximal therapeutic effects at a q4dx4 schedule (Francisco *et al*, 2003). Administration of ABVD or SGN-35 alone to L540cy tumour-bearing mice induced tumour regressions and significant TGD compared with control treatment (Fig 1A), resulting in 0/8 and 4/9 durable responses in ABVD and SGN-35-treated animals respectively. In contrast, combination of SGN-35 with ABVD resulted in 9/9 durable tumour regressions in all experimental animals (Fig 1A) and a statistically significant increase in TGD relative to each treatment arm alone (combination *versus* SGN-35: *P* < 0·0101, combination *versus* ABVD: *P* < 0·0001). Similarly, when treatment was initiated when tumours reached 300 mm^3^ volume, the combination of SGN-35 with ABVD significantly increased the TGD, resulting in 50% durable responses (5/10 animals; Fig. 1B). The delay in tumour growth induced by the combination treatment was highly significant relative to each individual treatment arm alone (combination *versus* SGN-35: *P* < 0·05, combination *versus* ABVD: *P* < 0·001; Fig. 1C).

**Fig 2:**
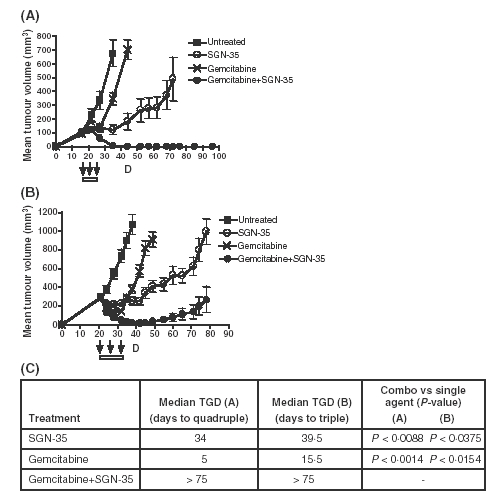
Antitumour activity of SGN-35 in combination with gemcitabine on subcutaneous L540cy HL tumours in SCID mice. SCID mice were implanted with L540cy HL cells in the right flank. Groups of mice (5–10/group) were untreated or received SGN-35 (1 mg/kg, q4dx3, i.p.) and/or gemcitabine (120 mg/kg, q4dx3, i.p.) when tumour size averaged approximately 100 mm^3^ (A) or 300 mm^3^ (B). The onset and duration of treatment is indicated by the bars within the figures. Bars within the graph represent standard deviation. (C) Median delay to a four- or threefold increase in tumour size (days) relative to untreated tumours are shown for individual treatment groups shown in panel A and B respectively.

Next, we studied the effects of combining SGN-35 with gemcitabine, a pyrimidine antimetabolite that inhibits DNA synthesis and is increasingly used for the treatment of relapsed and refractory HL patients because of its favourable safety and activity profile. For this purpose, mice were implanted with L540cy tumours and treated with SGN-35 and gemcitabine, either alone or combined. While single agent treatment led to significant delays in tumour growth, the combination of SGN-35 with gemcitabine enhanced the antitumour activity and induced durable responses in all animals (5/5, Fig 2A, combination *versus* SGN-35: *P* < 0·0088, combination *versus* gemcitabine: *P* < 0·0014). Improved activity in the combination treatment group was also noted when drug administration occurred when tumours reached a substantially larger size (300 mm^3^; Fig 2B). Similar to the ABVD experiment, combination treatment with gemcitabine resulted in a significant delay in tumour growth, which was more than additive (combination *versus* SGN-35: *P* < 0·0375, combination *versus* gemcitabine: *P* < 0·0154; Fig 2C) and frequently led to durable responses. Importantly, the combination of SGN-35 with other chemotherapeutic agents, such as vinorelbine, did not uniformly improve efficacy, suggesting that the effects were chemotherapy-specific (Fig S1). Finally, no significant differences in body weight loss or morbidity were noted in the ABVD and gemcitabine combination groups (Fig S2), indicating comparable tolerability in this experimental model. It is worth noting that SGN-35 does not cross-react with rodent CD30 and therefore, the tolerability comparison is based on the off-target toxicity of SGN-35. To determine the effects of combining SGN-35 with chemotherapy more conclusively, detailed toxicological studies in reactive species such as cynomolgus monkeys, will be required. To our knowledge, this is the first report demonstrating improved therapeutic benefit when combining ADCs targeting tumour-specific antigens with chemotherapeutic agents in preclinical models.

Better understanding of the mechanism causing the improvement in activity when combining SGN-35 with certain chemotherapy regimens may help to further refine the treatment. One possible cause could be drug interference with target expression. However, immunohistochemical staining of tumours did not reveal significant changes in CD30 expression in sections from tumours treated with either ABVD or gemcitabine, ruling-out this possibility (data not shown). Alternatively, differences between the cell types targeted by each class of compounds may account for improved activity. SGN-35 is directed towards neoplastic Reed Sternberg cells, which typically comprise only 0·1–1% of the total cell population within HL lesions (Bai *et al*, 2005). Chemotherapeutic agents, in addition to tumour cells, also target proliferating stromal cell infiltrates, which constitute the majority of the cell populations within HL lesions (Kuppers & Rajewsky, 1998). Thus, a model based on the reduction of neoplastic tumour cells by the ADC and a concomitant interference with supporting stromal cells by the chemotherapy may account for the increase in therapeutic activity. Secondly, differences in the mechanism of action between auristatin-based ADCs and chemotherapeutic compounds may help to explain the increase in efficacy. In support of this hypothesis, SGN-35 induces G2/M-phase growth arrest (Francisco *et al*, 2003), whereas gemcitabine blocks the cell cycle in S-phase (Zupi *et al*, 2005). ABVD is most commonly used for the treatment of HL (Duggan *et al*, 2003) and the individual compounds induce cell cycle arrest at all stages during cell division (Chabner *et al*, 2001). Auristatins, vinorelbine and vinblastine are cell cycle-specific agents, belonging to the vinca alkaloids that have slightly different mechanisms of action, partly as a result of the differences in their interaction with microtubule-associated proteins. Therefore, the increase in antitumour activity of SGN-35 in combination with gemcitabine and ABVD, but not vinorelbine, suggests that the efficacy improvements are specific for the type of cytotoxic compound and their mechanism of action. In conclusion, our findings provide a rationale for the design of clinical studies combining SGN-35 with chemotherapeutic regimens, such as ABVD and gemcitabine, with the goal to achieve improved efficacy, acceptable safety and reduced long-term complications in patients with HL.

## Supplementary material

The following supplementary material is available for this article online:

Fig S1 Antitumour activity of SGN-35 in combination with vinorelbine in a subcutaneous of L540cy HL tumours in SCID mice. SCID mice were implanted with L540cy HL cells in the right flank. Groups of mice (9–10/group) were either untreated or received SGN-35 (1 mg/kg, q4dx3, i.p.) and/or Gemcitabine (4 mg/kg, q5dx3, i.p.) when tumour size averaged approximately 100 mm^3^.

Fig S2 Mean percent body weight change of L540cy HL tumour bearing animals. (A) ABVD study. Groups of mice (9–10/group) were either untreated or received SGN-35 (1 mg/kg, q4dx3, i.p.) and/or ABVD [Adriamycin (0.75 mg/kg, q4dx3, i.v.), Bleomycin (6 mg/kg, q4dx3, i.p.), Vinblastine (0.01 mg/kg, q4dx3, i.p.) and Dacarbazine (15 mg/kg, q3dx4, i.p.)]. (B) Gemcitabine study. Groups of mice (5–10/group) were untreated or received SGN-35 (1 mg/kg, q4dx3, i.p.) and/or gemcitabine (120 mg/kg, q4dx3, i.p.). Onset of the treatments is labelled as day 0. The material is available as part of the online article from: http://www.blackwell-synergy.com/doi/abs/10.1111/j.1365-2141.2008.07146.x (This link will take you to the article abstract).

## References

[b1] Bai M, Papoudou-Bai A, Kitsoulis P, Horianopoulos N, Kamina S, Agnantis NJ, Kanavaros P (2005). Cell cycle and apoptosis deregulation in classical Hodgkin lymphomas. In Vivo.

[b2] Byrne BJ, Gockerman JP (2007). Salvage therapy in Hodgkin's lymphoma. Oncologist.

[b3] Chabner BA, Wilson W, Supko J, Beutler E, Lichtman MA, Coller BS, Kipss TJ, Seligsohn U (2001). Pharmacology and toxicity of antineoplastic drugs. Williams Hematology.

[b4] Duggan DB, Petroni GR, Johnson JL, Glick JH, Fisher RI, Connors JM, Canellos GP, Peterson BA (2003). Randomized comparison of ABVD and MOPP/ABV hybrid for the treatment of advanced Hodgkin's disease: report of an intergroup trial. Journal of Clinical Oncology.

[b5] Francisco JA, Cerveny CG, Meyer DL, Mixan BJ, Klussman K, Chace DF, Rejniak SX, Gordon KA, DeBlanc R, Toki BE, Law CL, Doronina SO, Siegall CB, Senter PD, Wahl AF (2003). cAC10-vcMMAE, an anti-CD30-monomethyl auristatin E conjugate with potent and selective antitumor activity. Blood.

[b6] Hamblett KJ, Senter PD, Chace DF, Sun MM, Lenox J, Cerveny CG, Kissler KM, Bernhardt SX, Kopcha AK, Zabinski RF, Meyer DL, Francisco JA (2004). Effects of drug loading on the antitumor activity of a monoclonal antibody drug conjugate. Clinical Cancer Research.

[b7] Klimm B, Schnell R, Diehl V, Engert A (2005). Current treatment and immunotherapy of Hodgkin's lymphoma. Haematologica.

[b8] Kuppers R, Rajewsky K (1998). The origin of Hodgkin and Reed/Sternberg cells in Hodgkin's disease. Annual Review of Immunology.

[b9] Santoro A, Bredenfeld H, Devizzi L, Tesch H, Bonfante V, Viviani S, Fiedler F, Parra HS, Benoehr C, Pacini M, Bonadonna G, Diehl V (2000). Gemcitabine in the treatment of refractory Hodgkin's disease: results of a multicenter phase II study. Journal of Clinical Oncology.

[b10] Schnell R, Borchmann P (2006). SGN-30 (Seattle Genetics). Current Opinion in Molecular Therapeutics.

[b11] Wahl AF, Klussman K, Thompson JD, Chen JH, Francisco LV, Risdon G, Chace DF, Siegall CB, Francisco JA (2002). The anti-CD30 monoclonal antibody SGN-30 promotes growth arrest and DNA fragmentation in vitro and affects antitumor activity in models of Hodgkin's disease. Cancer Research.

[b12] Younes A, Kadin ME (2003). Emerging applications of the tumor necrosis factor family of ligands and receptors in cancer therapy. Journal of Clinical Oncology.

[b13] Zupi G, Scarsella M, D’Angelo C, Biroccio A, Paoletti G, Lopez M, Leonetti C (2005). Potentiation of the antitumoral activity of gemcitabine and paclitaxel in combination on human breast cancer cells. Cancer Biology & Therapy.

