# Supplementary material for: Combination of the anti-CD30-auristatin-E antibody-drug conjugate (SGN-35) with chemotherapy improves antitumour activity in Hodgkin lymphoma
Source: Br J Haematol. 2008 May 8;142(1):69–73. doi: 10.1111/j.1365-2141.2008.07146.x (PMC2440525; doi:10.1111/j.1365-2141.2008.07146.x)
Supplement: Fig S1 — Antitumour activity of SGN-35 in combination with vinorelbine in a subcutaneous of L540cy HL tumours in SCID mice. SCID mice were implanted with L540cy HL cells in the right flank. Groups of mice (9–10/group) were either untreated or received SGN-35 (1 mg/kg, q4dx3, i.p.) and/or Gemcitabine (4 mg/kg, q5dx3, i.p.) when tumour size averaged approximately 100 mm3. [file bjh0142-0069-SD1.ppt]

## Slide 1
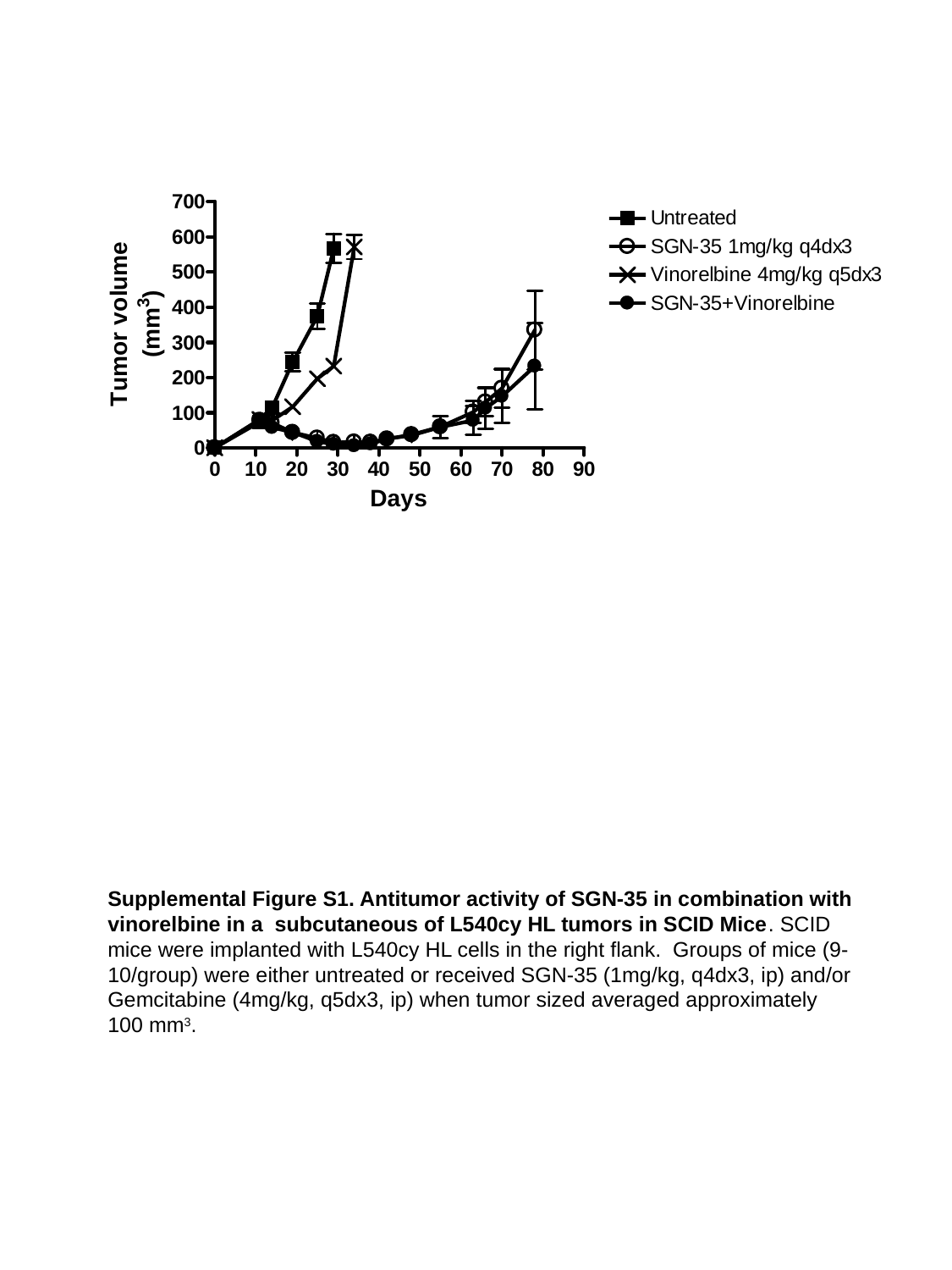

Supplemental Figure S1. Antitumor activity of SGN-35 in combination with vinorelbine in a subcutaneous of L540cy HL tumors in SCID Mice. SCID mice were implanted with L540cy HL cells in the right flank. Groups of mice (9-10/group) were either untreated or received SGN-35 (1mg/kg, q4dx3, ip) and/or Gemcitabine (4mg/kg, q5dx3, ip) when tumor sized averaged approximately 100 mm3.

## Slide 2
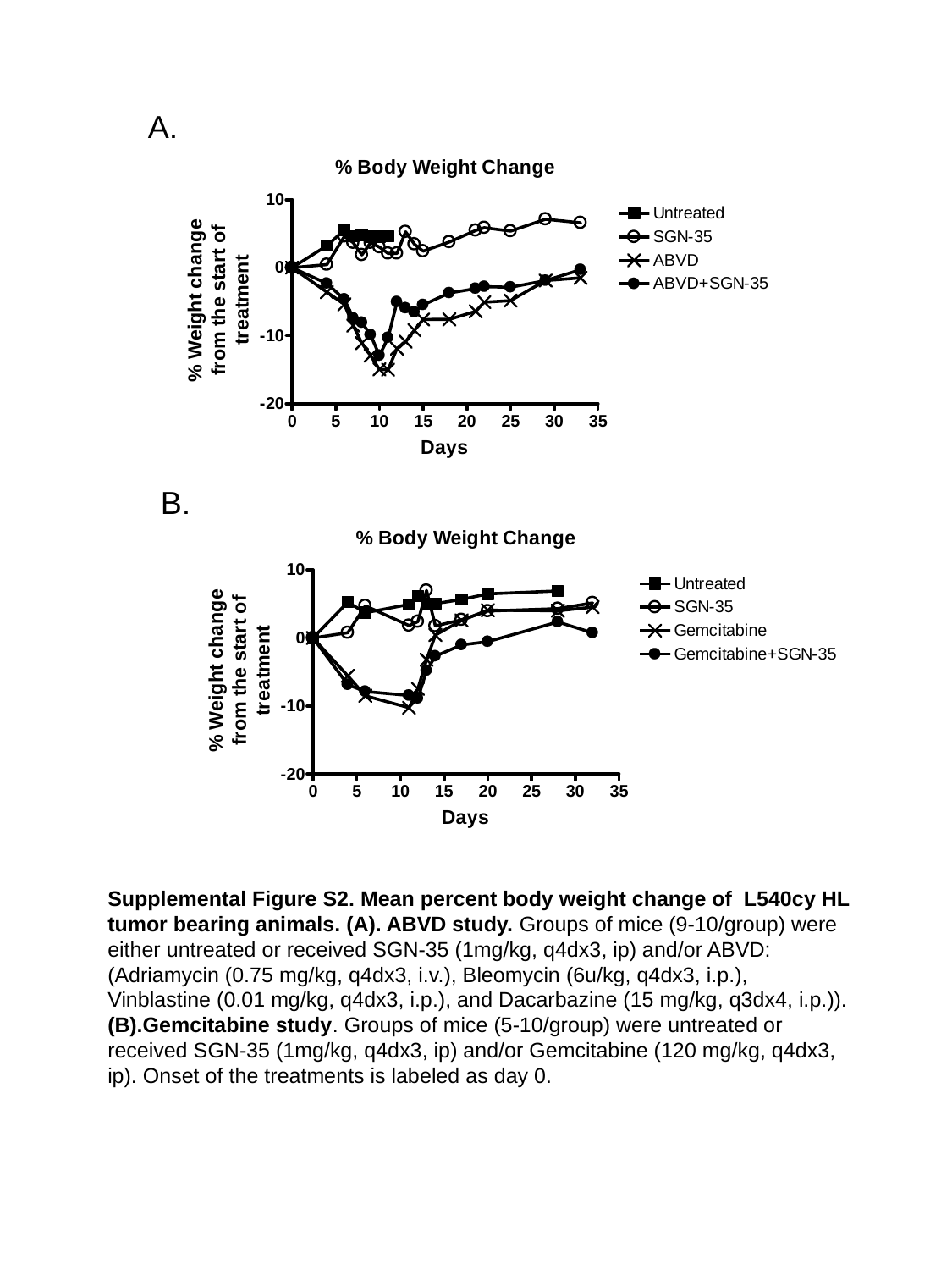

A.
B.
Supplemental Figure S2. Mean percent body weight change of L540cy HL tumor bearing animals. (A). ABVD study. Groups of mice (9-10/group) were either untreated or received SGN-35 (1mg/kg, q4dx3, ip) and/or ABVD: (Adriamycin (0.75 mg/kg, q4dx3, i.v.), Bleomycin (6u/kg, q4dx3, i.p.), Vinblastine (0.01 mg/kg, q4dx3, i.p.), and Dacarbazine (15 mg/kg, q3dx4, i.p.)). (B).Gemcitabine study. Groups of mice (5-10/group) were untreated or received SGN-35 (1mg/kg, q4dx3, ip) and/or Gemcitabine (120 mg/kg, q4dx3, ip). Onset of the treatments is labeled as day 0.
